# DPOAE Intensity Increase at Individual Dominant Frequency after Short-Term Auditory Exposure

**DOI:** 10.1155/2013/379719

**Published:** 2013-09-05

**Authors:** Judit Bakk, Tamás Karosi, Tamás József Batta, István Sziklai

**Affiliations:** Department of Otolaryngology and Head and Neck Surgery, Medical and Health Science Center, University of Debrecen, Debrecen 403, Hungary

## Abstract

Previous experiments suggested the possibility of a short-term sound stimulus-evoked and transient increase in DPOAE amplitudes. This phenomenon is possibly due to the complexity of the outer hair cells and their efferent control system and the different time scales of regulatory processes. A total of 100 healthy subjects ranging from 18 to 40 years of age with normal hearing and normal DPOAE values in the range of 781–4000 Hz were recruited in the study. Diagnostic DPOAE measurements were performed after short-term sound exposure. We proposed a 10 sec, 50 dB sound impulse as the most effective stimulus for clinical practice between 40 and 60 sec poststimulus time to detect the aforementioned transient DPOAE increase. We developed a procedure for detection of this transient increase in DPOAE by the application of a short-term sound exposure. The phenomenon was consistent and well detectable. Based on our findings, a new aspect of cochlear adaptation can be established that might be introduced as a routine clinical diagnostic tool. A mathematical model was provided that summarizes various factors that determine electromotility of OHCs and serves as a possible clinical application using this phenomenon for the prediction of individual noise susceptibility.

## 1. Introduction

The outer hair cells play a crucial role in the mammalian cochlea. These cells are part of a complex system that is necessary to detect low intensity sounds as well as to provide a self-defense against high intensity sounds [1]. In the mammalian cochlea, there is a complex mechanism, also known as cochlear amplification that provides the capability of detecting sounds of threshold intensity. Otoacoustic emission is also the result of active outer hair cell (OHC) motility, also known as electromotility [2–4]. Beyond the fast motility of OHCs (electromotility), these cells also exhibit an additional slow change in cell shape (slow motility). Slow motility is presented by cell shortening, which is assumed to play a protective role against loud sounds [5–9]. 

The slow motility of OHCs can modify the axial and lateral wall stiffness of cells decreasing the magnitude of their electromotile responses [7–9]. The slow motility of OHCs and the resultant cell stiffness changes can be considered as an intrinsic regulatory mechanism of OHCs. This mechanism is mechanically evoked, and it is independent from electromotility but depends on the presence and concentration of [Ca^2+^]_i_ and is also linked to the metabolic modification of cytoskeletal structure [7–10]. Furthermore, the axial and lateral wall stiffness determines the electromotility magnitude of OHCs that was particularly described by a mathematical model [9]. Decrease in magnitudes of electromotility can result in a measurable change in the otoacoustic emission [11].

In summary, the mechanically evoked increase in lateral wall stiffness and subsequent OHC shortening are intrinsic regulatory settings in cochlear amplification. This change is controlled by efferent neurotransmitters (acetyl-choline, Ach; gamma-amino-butyric acid (GABA)) that provide excitatory or inhibitory neuronal feedback. These neurotransmitters temporarily decrease the lateral wall stiffness. In contrast, the persisting mechanic stimulation results in increased lateral wall stiffness and OHC shortening, which overcomes the initial cell stiffness decrease due to the neurotransmitters. The summation of these antagonistic processes will result in measurable increase of OAE magnitudes. A sound stimulation of appropriate duration and intensity will evoke a transient increase in the otoacoustic emission. This phenomenon is tied to the intrinsic stiffness-regulated mechanism of OHCs.

The steady-state axial and circumferential stiffness of OHCs is regulated by a complex Ca^2+^-dependent phosphorylation-dephosphorylation mechanism that modulates the structure of the subcortical cytoskeleton [7–10, 12]. The transient otoacoustic emission intensity increase induced by sound stimulation can be established as a sensitive indicator of the changes in metabolism and operation of OHCs. This assumption has already been highlighted by Kiss et al., who studied the changes in human otoacoustic emission intensity after a 3 minutes exposure by wide-band noise and pure tone [13]. They documented a distortion product OAE (DPOAE) intensity increase also at low and high frequencies (500, 625, 781, 1000, 3187, 4000, and 5031 Hz) and a decrease at medium frequencies (1250, 1593, 2000, and 2531 Hz). 

Abel et al. reported similar observations in mongolian gerbils during contralateral acoustic stimulation (white noise stimulus intensity range of 10–70 dB, SPL) [14]. Altogether 12 out of 14 animals displayed increased *f*2 − *f*1 DPOAE amplitude, while in the rest of subjects it was decreased. Simultaneously, the 2*f*1 − *f*2 DPOAE amplitude did not increase or only slightly increased. These findings and other observations published about the adaptation of DPOAE suggest that the function of OHCs and mechanical control in the cochlea can be monitored [15–17].

The aim of the present study is the characterization and mathematical description of the potential clinical application of transient increase in DPOAE intensity due to auditory exposure in humans by the application of a standard DPOAE measurement setup.

## 2. Materials and Methods

The measurements were performed on one hundred healthy young volunteers ranging from 18 to 40 years of age with normal hearing and normal DPOAE values in the range of 781–4000 Hz. The Institutional Ethical Committee accepted this study. Subjects gave their written informed consent to our study. This study was carried out according to the declaration of Helsinki. All subjects had normal tympanogram (A-type) and normal stapedial reflex in both ears. The middle ear resonance frequencies varied between 781 and 2000 Hz, with an average of 1172.5 Hz ± 269.9 Hz. Before each measurement, the subjects were isolated for at least half an hour in a soundproof environment, and the measurements were also performed in a soundproof room. DPOAE measurements were performed using a GSI 60 instrument (Grason Stadler, Eden Prairie, USA), which generated two primary frequency tones, 2*f*1 − *f*2 with a stimulus frequency separation of *f*1/*f*2 = 1.2. Intensity of the custom stimulus was 70 dB SPL at both frequencies. The DPOAE was recorded by manually scanning the 781–4000 Hz frequency interval focused on the pure tone audiometric test frequencies before and directly after the auditory exposure. To avoid the excitation of the adjacent frequencies in the cochlea, we scanned according to the following order: 1593 Hz–4000 Hz–1000 Hz–3187 Hz–781 Hz–2000 Hz. DPOAE was measured immediately after the stimulus at each frequency. The auditory exposure (pure tone and wide-band noise) was added via earphones both ipsilateral and contralateral application, respectively. The frequencies of pure tone stimuli were close to the frequency of DPOAE elicitor tones *f*2 [17]. The effect of different intensity sound exposures on the changes of DPOAE magnitude was studied with pure tone sound impulses of 10 sec in duration with various intensities between 20 and 80 dB SPL (in 10 dB increments). The best DPOAE intensity responses were obtained by using 50 dB SPL sound impulses of various durations (3, 5, 10, 30, and 300 sec). To investigate the decay of the response to a 10 sec, 50 dB SPL pure tone stimulus, we measured DPOAE responses at 30 sec intervals after the stimulus at the characteristic frequency (i.e., where the greatest intensity response is measured) for 300 sec. Between any two sessions, the subjects rested for a minimum of 30 minutes in silence. The change in DPOAE intensity (ΔDPOAE) refers to the difference between the poststimulus and the initial DPOAE magnitude throughout this paper.

## 3. Results

### 3.1. DPOAE Changes Evoked by Different Forms of Sound Stimuli

**Figure 1:**
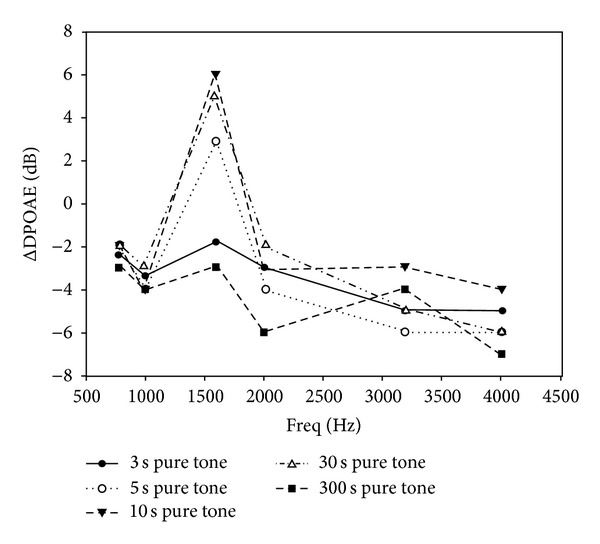
ΔDPOAE at the characteristic frequency after 50 dB SPL, 5–10–30–300 sec, pure tone stimulus. The characteristic frequency (see the text) of the subject no. 1 is 1593 Hz. The most effective stimulus duration is 10 sec.

**Figure 2:**
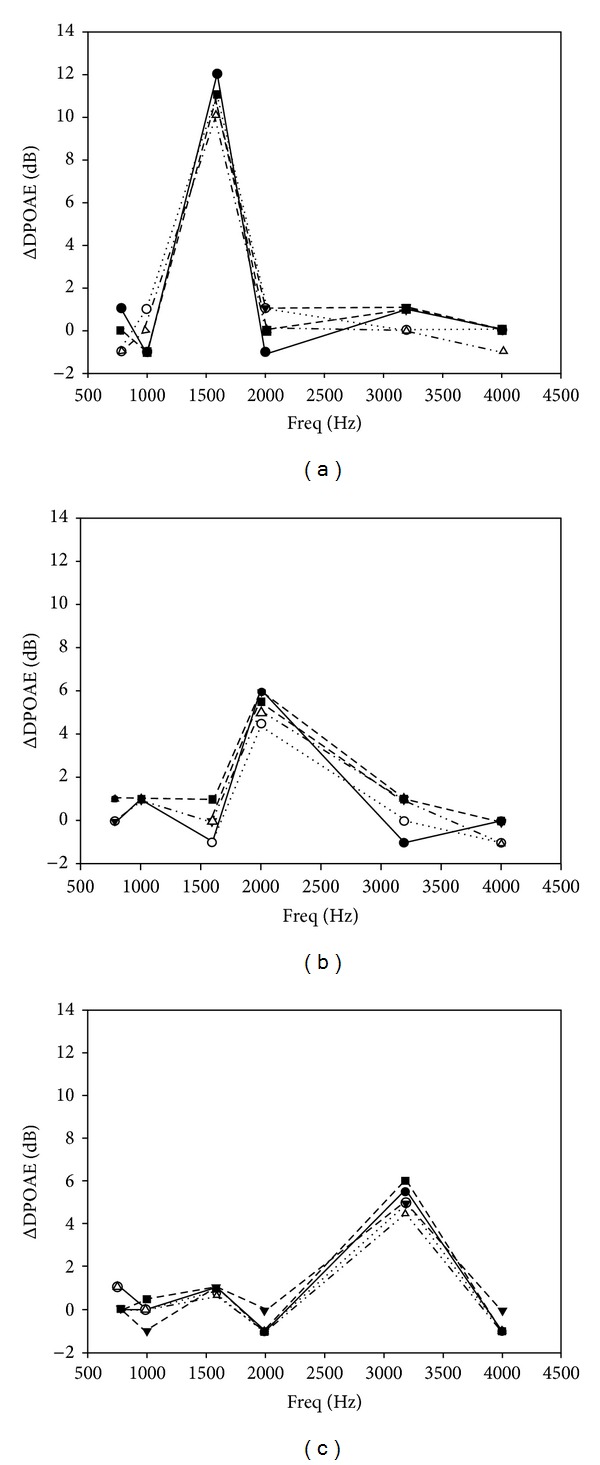
The reproducibility of the sound impulse induced transient increase in DPOAE intensity. Different lines and symbols show the results of how 10 sec, sequentially applied, 50 dB SPL pure tone stimuli evoked changes in the DPOAE magnitude at 5 different times in each subject. The graphs ((a), (b), (c)) demonstrate different subjects (no. 2, 5, 7) with different characteristic frequencies ((a): 1593 Hz, (b): 2000 Hz, (c): 3187 Hz).

**Figure 3:**
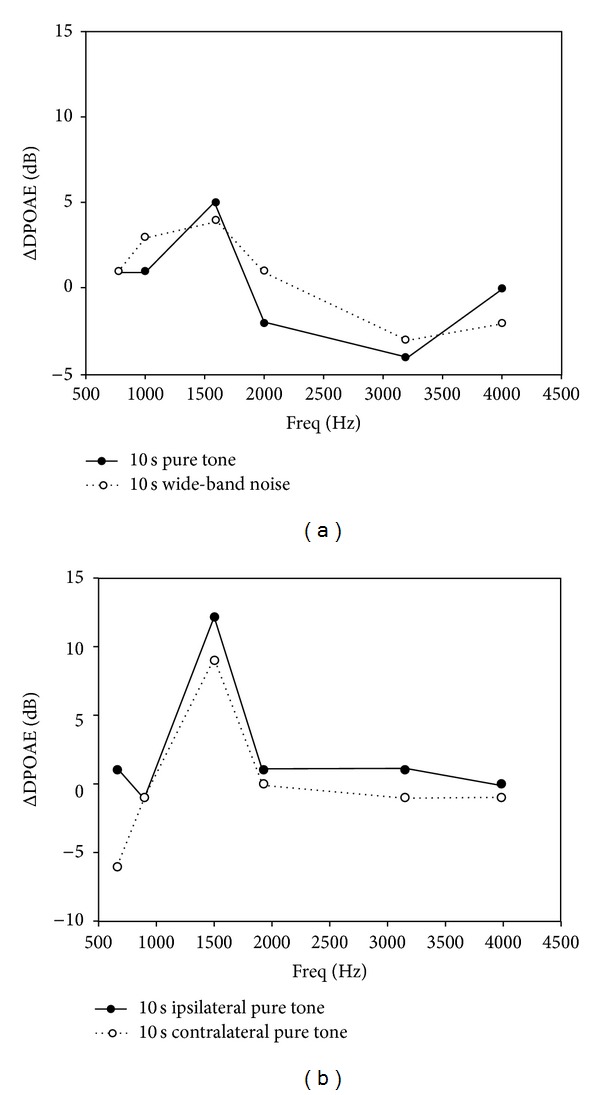
(a) Typical ΔDPOAE curves in the frequency function after a single 10 sec, 50 dB SPL pure tone or wide-band noise stimulus. (b) Typical ΔDPOAE frequency curves after a single 10 sec, 50 dB SPL pure tone, ipsilateral, and contralateral stimulus. The characteristic frequency (see the text) of the presented subject (no. 14) is 1593 Hz.

A single 10 sec pure tone evoked a transient increase in the DPOAE magnitude. The DPOAE magnitude increase was frequency- and subject-specific. The frequency of the greatest DPOAE intensity increase (individual dominant frequency (IDF)) varied individually, but each subject demonstrated a clear increase in DPOAE intensity at the characteristic frequency (CF) (Table 1). The IDF was independent from the sound intensity and the duration of sound stimulus between 5 and 300 sec (Figure 1, Table 1). There was a marked DPOAE intensity increase at the frequencies adjacent to the IDF in 35% of the cases; this intensity increase was, however, 50–70% lower than that at the CF. The IDF or the change in the DPOAE intensity was consistent and reproducible, individually (Figure 2). This frequency was independent from the resonance frequency of the middle ear itself. Wide-band noise stimulus (50 dB, 10 sec) also evoked an exclusive DPOAE intensity increase at the IDF. This change was smaller than the response to a pure tone stimulus (Figure 3). At the frequencies adjacent to the IDF, some ΔDPOAE increase was consistently observed. Ipsilateral and contralateral sound exposures resulted in similar responses. Contralateral sound exposure, however, induced slightly less increase than the ipsilateral one (Figure 3). At IDFs, the ΔDPOAE showed an average of 7.1 dB ± 2.31 dB (±SE) after the most effective 10 sec, 50 dB pure tone stimulus and corresponded to a 20–500% increase in intensity. The ΔDPOAE increased with increasing stimulus intensity in the range of 20–50 dB, peaks at 50 dB, and decreased at higher intensities (Table 2).

### 3.2. The Effect of the Duration of Pure Tone Sound Stimulus on ΔDPOAE

A single 3 sec, 50 dB sound stimuli resulted in a DPOAE intensity decrease. A single 50 dB pure tone stimulus with 5, 10, or 30 sec duration caused a DPOAE intensity increase. ΔDPOAE varied with the duration of the sound stimulus, but the IDF remained unchanged. The increase in the DPOAE was smaller after a 5 sec stimulus; however, the maximum response was obtained using a 10 sec stimulation time (Figure 1). After a relatively long (5 min) 50 dB pure tone stimulus, DPOAE responses decreased in each subject at each frequency, similar to previous reports in the literature (Figure 1) [18]. In agreement with our previous *in vitro* experiments, the 10 sec stimulus was found to be the most effective. In contrast, shorter or longer stimuli decreased the response magnitude (Figure 1). A 5 sec stimulus was probably not long enough to evoke the efferent neurotransmitter-related reduction in lateral wall stiffness. The 30 sec stimulus increased these stiffness characteristics by activating the regulatory stiffness response and reducing the DPOAE magnitude as a consequence of adverse processes. A five-minute auditory exposure decreased the otoacoustic emission at all frequencies. The DPOAE decrease after a 3 sec sound stimulus might confirm the two-phase efferent effect that was previously assumed in the literature [19–21].

### 3.3. The Decay of the DPOAE Intensity Increase Evoked by Pure Tone

The decay of the 50 dB pure tone evoked ΔDPOAE differed across individuals and varied between 3 and 5 minutes. 

## 4. Discussion

OHCs are assumed to be the active elements of the cochlear energy feedback system or cochlear amplifier and serve simultaneously as a defense against high intensity sounds [1, 4]. OHCs are generally considered to provide the high sensitivity and fine-tuning in the mammalian organ of Corti. Efferent innervation of the OHCs is reported to modify the efficacy of the amplifier mechanism [20–23].

DPOAE is a widely used examination method in the clinical practice for the monitoring of OHCs' function (the cochlear amplifier). Typically 2*f*1 − *f*2 (*fdp* = *f*1 − [*f*2 − *f*1] = 2*f*1 − *f*2) stimulus is used and plotted in the function of frequency as *f*1 and *f*2 sweep along the frequency range of interest [24]. Although some functional changes can be detected in the cochlear amplifier by standard application of DPOAE, other functional impairments, however, cannot be followed by 2*f*1 − *f*2 stimuli. Recently Kössl et al. described a DPOAE increase mediated by the medial olivocochlear projections (medial olivocochlear bundle (MOC)). This can be measured during acoustic stimulation using quadratic DPOAE *f*2 − *f*1 method [14, 17]. This phenomenon is highly connected to the duration of the acoustic stimulus. Furthermore, detection by standard 2*f*1 − *f*2 DPOAE usually gives uncertain results. It has been suggested that this phenomenon derives from the nonlinear behavior of the stereocilial bundles of OHCs and efferent projections may directly affect the operating points of OHCs.

In this study we report a similar DPOAE increase, which is elicited by a relatively long acoustic stimulus (more than 5 seconds). It takes relatively long time to appear after the acoustic stimulation (approximately 3 minutes), and it is detectable using standard 2*f*1 − *f*2 stimulation. The different behavior of the two DPOAE increasing phenomenon calls the attention for modeling different background mechanisms.

The activation of the medial olivocochlear neural projections opens the postsynaptic ion channels that results in depolarization of OHCs (slow medial olivocochlear effect, 10–100 s) [19–21, 25]. In turn, this effect enhances cochlear amplification, auditory sensitivity, and otoacoustic emission magnitude due to the electromotile activity increase in OHCs [7–9, 23]. The decrease in the axial and lateral wall stiffness parameters is a consequence of increasing intracellular [Ca^2+^]_i_ levels in OHCs that is secondary to the sustained effect of efferent neurotransmitters [7, 26, 27]. Another control on the axial and lateral wall stiffness of OHCs is the mechanically stimulated slow cell motility and the regulatory stiffness response of OHCs [7]. This can drastically increase the stiffness of OHCs and consequently reduce the efficiency of cochlear amplification [8, 9]. 

The regulatory stiffness response, being an intrinsic setting of the OHC lateral wall stiffness, peaks at 40–50 sec after either a mechanical or an auditory stimulus. The influence of ACh and GABA on the electromotility of OHCs develops within 10 sec [28]. This result suggests that the sensitivity of cochlea is reduced in the short time interval between the different time scales of these two regulatory mechanisms. This short interval is presented by a unique phenomenon of transient increase in the DPOAE magnitude after a short-term sound stimulus. Long-term acoustic stimulation allows time for the stiffness to increase the cell response enough to compensate or reverse the effect of the previous decrease in lateral wall stiffness. Finally, this balance results in a net decrease in DPOAE intensity.

For modeling the DPOAE intensity increase phenomenon after short-term auditory exposure, we suppose that the relationship between the electromotility of OHCs and otoacoustic emission in the external auditory canal is similar to the extracochlear electrically evoked otoacoustic emission (EEOAE). EEOAE is characterized by a linear relationship between the magnitude of electric stimulus and the intensity of otoacoustic emission in the external auditory canal [11]. The electromotility of OHCs exponentially depends on the lateral wall stiffness [8, 9]. OHCs respond to mechanical and sound stimuli by shortening in parallel to the increase in lateral wall stiffness. The time course of these processes can be described by a Boltzmann-like function. The OHC length and lateral wall stiffness return to resting values after a mechanical stimulus with similar dynamics [7–9]. The lateral wall stiffness decreases in the presence of efferent neurotransmitters, whereas the stiffness increases in response to a mechanical and sound stimulus [23]. The lateral wall stiffness reducing effect of neurotransmitters develops faster (~10 sec) compared to the increased stiffness due to mechanical or sound stimulus (~50 sec) [7, 23]. Thus, after a sound stimulus that triggers both processes, there may be a transient increase in electromotility and otoacoustic emission. This increase is compensated by the delayed OHC lateral wall stiffness response. The lateral wall stiffness of OHCs is reduced by 20% in the presence of efferent neurotransmitters, whereas the sound-induced lateral wall stiffness increases by 30% simultaneously [23]. Borkó et al. described the exponential OHC electromotility dependence on lateral wall stiffness in this region using a linear function [8, 9]. The slope is ~1.7 mV/nN/*μ*m. The correlation between electromotility and otoacoustic emission is also linear with a slope of ~1.6 *μ*m/dB [11]. The difference between the baseline otoacoustic emission (OAE_0_) and the theoretical emission (OAE_1_) due to neurotransmitters and mechanical or sound stimulus is 230%. The theoretical otoacoustic emission increase should be 6.5 dB, which is quite close to the measured value of 6.7 dB [23]. The difference between OAE_0_ and theoretical emission (OAE_2_) after a mechanically induced cell response is 250%.

The two processes run parallel to each other (albeit on a different time scale), and thus the magnitude of otoacoustic emission at each moment is determined by their combined effect. In conclusion, we can indirectly measure changes in the lateral wall stiffness parameter by measuring changes in DPOAE amplitudes due to the linear relationship between otoacoustic emission and lateral wall stiffness. 

We provide the following explanation for the mechanism of these changes. Efferent neurotransmitters increase the intracellular [Ca^2+^]_i_ concentration. This increase shifts the phosphorylation-dephosphorylation balance of cytoskeletal proteins. The protein deformation results in decreased lateral wall stiffness. This decrease is exponential in time [23]. The slow OHC motility caused by a mechanical stimulus (sound) and the simultaneous increase in the lateral wall stiffness can be fitted by a Boltzmann-like function [7]. Consequently, following a sound stimulus that activates both the slow and fast motility of OHCs, the time course of the otoacoustic emission change can be described by the difference of an exponential association and a Boltzmann-like function:

(1) $$\begin{array}{c} f\left(t\right)=a\left(1-e^{-bt}\right)-\frac{c}{1+e^{-\left(t-t_{0}\right)/d}}, \end{array}$$

where *a* is the difference of the maximal otoacoustic emission and the baseline value; *b* is the time constant of the effect of efferent neurotransmitters; *c* is the maximum decrease of otoacoustic emission due to an increase in cell stiffness in response to slow motility; *d* is the time constant of the mechanical cell response; *t* is time; *t*_0_ is the half-life of the slow motility; and *e* is the base of the natural logarithm.

According to the fact that increase in intrinsic regulatory stiffness, lateral wall stiffness, and electromotility are all linearly related to the otoacoustic emission, their time courses are also characterized by similar functions. 

**Figure 4:**
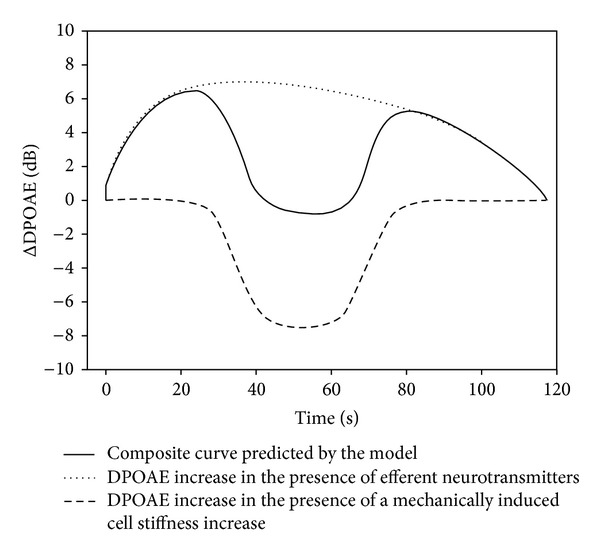
Model curve according to the studies of Ren et al., Batta, and Borkó. The model describes the DPOAE change in the simultaneous presence of efferent neurotransmitters and the mechanically induced increase in OHC lateral wall stiffness. Continuous line shows the curve calculated by the model (see (1)). The dotted line shows increase of DPOAE in the presence of efferent neurotransmitters (first part of (1)). Dashed line indicates decrease of DPOAE in the presence of the mechanically induced increase in stiffness (second part of (1)).

**Figure 5:**
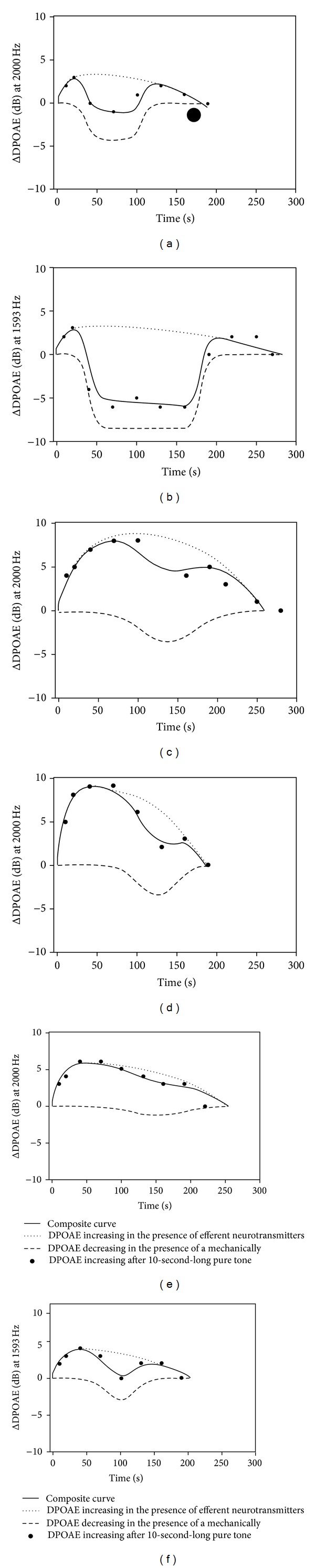
Decay of the DPOAE magnitude increase evoked by 50 dB SPL pure tone in six different subjects. Different tones demonstrate the DPOAE change at the characteristic frequencies. Data points are the measured ΔDPOAE values, and the solid line represents the mathematical model-predicted values. Continuous line shows the curve calculated by the model (see (1)). The dotted line indicates the increasing DPOAE in the presence of efferent neurotransmitters (first part of (1)). Broken line shows the decrease of DPOAE in the presence of the mechanically induced increase in OHC lateral wall stiffness (second part of (1)).

Borkó et al. found that cell responses to a mechanical stimulus develops and decays with similar characteristics. We assumed that the efferent neurotransmitter-induced changes in lateral wall stiffness behave similarly. The time course of DPOAE intensity changes due to appropriate sound stimulation that can be predicted by the mathematical model (Figure 4), which was developed on the basis of *in vitro* experiments [23]. The model shows exact fitting to discrete data points obtained from the present human study. The measured human otoacoustic emission values show substantial individual variability (Figure 5). 

The magnitude and frequency of OAE increase show individual differences and depends on the intensity of the sound stimulus (Figure 3, Tables 1 and 2). The OAE magnitude dependence on the stimulatory sound intensity is probably due to the activation of ACh-GABA mediated efferent nervous system. ACh and GABA may cause a sound-intensity-dependent OAE magnitude increase in response to the low and medium intensity sounds. Sound stimuli of higher intensity work by increasing the regulatory stiffness of the lateral wall of OHCs. This action overcomes as the opposite effect of ACh and GABA; therefore, the emission amplitude decreases (Table 2).

The time-related peak ΔDPOAE (30–60 sec) and decay (3–5 min) are determined by two regulatory mechanisms: (1) intrinsic lateral wall stiffness increase and (2) neurotransmitter-controlled stiffness of OHCs. These two processes start simultaneously, but the intrinsic mechanism works slower. The OHC lateral wall stiffness-reducing effect of neurotransmitters occurs faster (~10 sec) and lasts longer (at least 2 min) than the cell stiffness changes due to a sound stimulus (~50 sec) [7]. 

A possible explanation for the existence of characteristic frequency is the tonotopic distribution of ACh and GABA receptors that corresponds to the tonotopic location of OHCs in the cochlea [23, 29–31]. On the other hand, this explanation can be supplemented by the exponential relationship between the efferent neurotransmitter receptor activity and the OHC electromotility magnitude [8, 9, 23]. Former studies suggest that mammalian cochlea might be characterized by two different distribution patterns of ACh and GABA receptors: (1) a tonotopic increase in ACh receptor density from the helicotrema to the basilar turn and (2) a tonotopic increase in the numbers of GABA receptors towards the opposite direction [23, 32–34]. In contrast to this hypothesis, a different pattern of distribution of ACh and GABA receptors was also reported [31]. This study describes that cochlear regions displaying the highest receptor density are related to the mid-range frequencies. This distribution pattern might explain that characteristic frequencies are linked to the middle cochlear region. The inverse distribution pattern of Ach and GABA receptors could give a potential explanation for the emission increase in the mid-range frequencies. The combined effect of ACh and GABA on the electromotility of an isolated OHC can be calculated as a weighed geometric mean of the numbers of ACh and GABA receptors [23]. The greatest increase of the OHC electromotility and related DPOAE magnitude increase due to the activation of the efferent feedback can be expected in those cochlear regions that are characterized by equalized numbers of ACh and GABA receptors. The individual differences in characteristic frequencies may be due to different receptor distributions across individuals. At this time, no data are available about the receptor distributions in the human cochlea. Our previous *in vitro* and present *in vivo* observations require further morphologic and immunohistologic examinations in the future in order to confirm or to confute this hypothesis.

In summary, the adaptation process of DPOAE is composed of three well-differentiated phases: an early, a transient, and a late phase. The early phase is composed of two episodes: the previously described fast and slow adaptation [15]. The time interval of the fast adaptation is about 70 msec, and it is presented as a decrease in the DPOAE intensity [15]. Duration, magnitude, and tendency (i.e., stagnation, slight decrease, or increase of DPOAE intensity) of the slow adaptation of DPOAE intensity are individually different in humans [15]. In agreement with the results of *Kim*, team of *Kössl *reported that the time interval of slow adaptation is exerted between 1.5 and 10 seconds [14–16]. The actions in this phase of DPOAE adaptation are produced by the MOC system mediated operating point shift of OHCs, while the transient and late phase is generated by the stiffness change of OHCs' lateral wall. The transient phase of DPOAE adaptation is a complex intensity-time function, which is produced by the poststimulus OHC stiffness increase due to the intrinsic regulatory stiffness response and by the efferent control resulted OHC stiffness decrease (Figure 4). In the late phase of DPOAE adaptation, intrinsic regulatory stiffness response-mediated lateral wall stiffness increase in the OHCs overgrows the efferent neurotransmitter-mediated decreasing process of lateral wall stiffness resulting in DPOAE intensity decrease.

In conclusion, analysis of DPOAE intensity-time function after a single sound stimulus makes it possible to extract the two simultaneous regulatory mechanisms. This method has sufficient sensitivity and specificity for the *in vivo* measurement of the electromotility of OHCs. Our results suggest that clinical examinations are supposed to perform by the application of a 10 sec-long 50 dB pure tone sound stimulus. The ideal time interval for detecting DPOAE increase is between 40 and 60 sec after the stimulus. This method is well reproducible, reliable, and cheap and provides a “window” on the cochlear amplifier. Its standardization and clinical introduction might contribute to the evaluation of individual noise susceptibility. As a potential clinical test it can exhibit the functional cooperation between the MOC system and the organ of Corti.

## Figures and Tables

**Table 1:** Distribution of subjects disposed by individual dominant frequency (IDF).

IDF of DPOAE amplitude increase	781 Hz	1000 Hz	1593 Hz	2000 Hz	3187 Hz	4000 Hz

Number of subjects	22	19	28	12	14	5

**Table 2:** DPOAE changes in the function of stimulus intensity.

Intensity of the stimulus (dB, SPL)	Amplitude of DPOAE increase (dB); average ± SE (*n* = 28)^1^
20	4.75 ± 0.89
30	5.25 ± 0.99
40	6.75 ± 1.2
50	6.875 ± 1.299
60	5.5 ± 1.039
70	4.625 ± 0.87
80	3.875 ± 0.73

^1^ΔDPOAE increases in the function of stimulus intensity in the range of 20–80 dB, develops at 50 dB, and decreases thereafter. The measurements were recorded by using a characteristic frequency of 1593 Hz (*n* = 28, means ± standard error).
